# Continuous Jetting of Alginate Microfiber in Atmosphere Based on a Microfluidic Chip

**DOI:** 10.3390/mi8010008

**Published:** 2017-01-04

**Authors:** Junyi Zhao, Wei Xiong, Ning Yu, Xing Yang

**Affiliations:** 1 Department of Precision Instruments, Tsinghua University, Beijing 100084, China; zhao-jy15@mails.tsinghua.edu.cn (J.Z.); ningyuneo@gmail.com (N.Y.); 2 The State Key Laboratory of Precision Measurement Technology and Instrumentation, Tsinghua University, Beijing 100084, China; 3 Key Laboratory of Advanced Reactor Engineering and Safety of Ministry of Education, Collaborative Innovation Center of Advanced Nuclear Energy Technology, Institute of Nuclear and New Energy Technology of Tsinghua University, Beijing 100084, China; xwthu@tsinghua.edu.cn

**Keywords:** microfluidic chip, alginate microfiber, jet, nozzle, sheath flow, tissue engineering

## Abstract

We present a method based on a microfluidic chip that produces continuous jetting of alginate microfiber in the atmosphere to facilitate its collection and assembly. Through the analysis of the factors influencing the microfiber jetting, the principle and some microfluidic chip design criteria are discussed. A special nozzle is designed near the chip outlet, and deionized water is introduced into the microchannel through the nozzle to increase the flux and thus to prevent drop formation around the outlet which impedes the continuous jetting of microfiber. The experiments have reported the effectiveness of the proposed structure and shown that the introduction of sheath flow promotes the stability of the flow field in the microchannel and does not affect the morphology of microfiber. Simulations of velocity and pressure distribution in the microchannel are also conducted. Further, the jetting microfibers are collected and assembled into various 3D complex fiber-based macroscopic structures through patterning or reeling. Since the proposed structure is rather simple and can be easily integrated into other complex structures without adding more soft-lithographical steps, microfibers with various morphology and function can be synthesized and collected in a single chip, which can be applied to various fields, such as tissue engineering, biotechnology, and drug discovery.

## 1. Introduction

Microscale hydrogel fibers have shown great potential in many fields, such as tissue engineering, biotechnology, and drug discovery [1,2,3]. Microfluidic spinning through the fast chemical reaction between central and sheath flows has become an effective method for the fabrication of hydrogel fibers. The frequently used materials for chemical reaction, including alginate [4,5,6,7], chitosan [8,9,10], and poly(lactic-co-glycolic acid) (PLGA) [11,12], are biocompatible, biodegradable and nontoxic. The formation process is also performed under a mild atmosphere without high pressure or voltage, and thus, it has no adverse effect on biomaterials, such as cells and proteins. Extensive research has been conducted for the microfiber synthesis. Microfibers with diverse shapes—including cylindrical [7,8,13], flat [14,15], and hollow [16,17,18]—have been fabricated through various microfluidic chips. Moreover, multicomponent fiber with hybrid structure, like Janus fiber or shell fiber, can be fabricated [16,19,20,21]. These fibers have been used to encapsulate live cells [20,21,22,23,24,25], or alternatively, used as scaffolds to guide cell growth [11,14,21]. Many studies have shown that the cells exhibit a high survival rate and even maintain their intrinsic morphology and function in these structures. Furthermore, using these cell-laden hydrogel microfibers as a bottom-up element, complex 3D cellular architecture can be constructed [13,16,22,26,27], which can be applied for drug testing [8,24,28] and implantation [22,23,29].

However, some challenges during the collection and assembly of microfibers are still present. Take alginate fibers for example, the calcium chloride (CaCl_2_) aqueous solution as the outer flow and the sodium alginate (NaAlg) aqueous solution as the inner flow are introduced into the laminar flow microfluidic chip. When the two fluids contact, the alginate flow starts to solidify into fiber because of the chemical reaction between the diffused Ca^+^ and NaAlg. Although the fiber can be generated continuously in this method, it cannot smoothly jet from the outlet if the chip is placed in the air, because the redundant solution that has not solidified into fibers starts to form a drop around the outlet. The drop is retained by the surface tension force, until it becomes sufficiently large to overcome the surface tension force and to fall from the microchannel. During a drop accumulation, the continuously generated alginate fiber is confined in the drop, and thus the fiber cannot be easily collected and assembled into certain shapes, which is really detrimental to their applications in the field of tissue engineering and 3D print, etc. Also, the periodic accumulate-fall down process can cause a non-negligible perturbation and even clog the microchannel, which has unfavorable effects on fiber formation. To address this problem, the outlet of the microfluidic chip is usually immersed vertically into the CaCl_2_ solution to prevent drop formation and enable the generation of a stable jet of fiber. However, this method is also inconvenient because fiber collection and assembly processes need to be undertaken in a liquid environment. Few studies have been conducted on the jetting of microfibers in the atmosphere.

Considerable research has been conducted on a microfluidic liquid jet system based on hydrodynamic focusing using high pressure gas sheath or liquid flow [30,31,32] and these studies provide inspiration for us to solve the microfiber jetting problem. Still, these techniques cannot be directly used in the jetting of microfiber, for not considering the microfiber’s formation process through chemical reaction.

Herein, a simple microfluidic device applied for continuous jetting of alginate fibers is reported. Through soft lithography technology, a poly (dimethyl siloxane) (PDMS) microfluidic device with a specially designed nozzle is fabricated. Deionized water (DI water) is introduced into the microchannel around the outlet to increase the flux in order to avoid drop formation, and thus the fiber is continuously jetted from the microchannel into atmosphere. The flow rate of the DI water has little influence on the diameter of the microfiber. Furthermore, the DI water facilitates the retention of the stability of microchannel stream field. Since the proposed microfluidic device is structurally simple, involving just one lithography step in the fabrication process, and the replica mold can be used repeatedly, this method is easy and cost-effective. Notably, this nozzle structure can be easily integrated into all microfluidic structure previously proposed for synthesizing diverse microfibers, without complicating the production steps of microfluidic chip, and facilitating microfiber collection and assembly. Therefore, it can be applied to various situations where fiber-based macroscopic structures require precise assembly processes, such as weaving, knitting, reeling, and patterning.

## 2. Experimental

### 2.1. Materials

NaAlg powder purchased from Alfa Aesar Chemical Co., Ltd. (Tianjin, China) was dissolved in DI water with a mass fraction of 1.0 wt %. CaCl_2_ provided by J&K Scientific Co., Ltd. (Beijing, China) had an aqueous concentration of 20 g/L. Rhodamine B purchased from J&K Scientific Co., Ltd., was added to the NaAlg solution to facilitate observation under an optical microscope. The 184 silicone elastomer, which was used to make the PDMS device, was obtained from Dow Corning Co., Ltd. (Shanghai, China). All other chemicals were of analytical grade.

### 2.2. Principle and Structure Design

**Figure 1:**
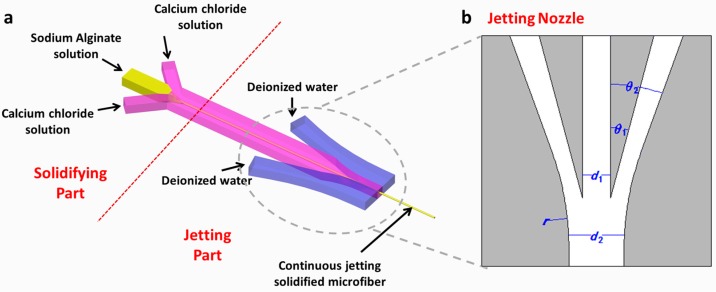
(**a**) Schematic diagram of channel flow; (**b**) Parameters of the jetting nozzle.

The liquid coming out from the microchannel can be analogous to the flowing water from the faucet. When the tap is slightly turned, a series of distinct drops forms, while when the faucet is completely opened, a steady stream can be observed. Similarly, when the liquid in the channel flows slowly, the liquid tends to be held around the outlet because of surface tension and its volume becomes larger constantly as more liquid flows out. The momentum of accumulated liquid mainly depends on the surface tension force, gravity and inertial force. When the volume of the liquid is sufficient to overcome the surface tension force, a drop forms and then falls down. When the flow rate of the liquid in the microchannel gradually increases, the period of drop formation and falling accelerates, and ultimately the liquid will jet from the outlet. This transition from dripping to jetting can be characterized by Weber Number [33,34]:

(1) $$We=\frac{\rho {V_{0}}^{2}D}{\sigma },$$

where ρ and σ represent the density and surface tension of liquid respectively, D represents the characteristic length of channel, and $V_{0}$ represents the velocity of liquid at the outlet. Jetting appears when the Weber number reaches the critical value, that is, the velocity reaches the critical value. According to the above analysis and principle, in order to avoid the dripping state around the outlet of the chip and to enable the stable jetting of microfiber, a microfluidic channel consisting of solidifying and jetting parts was proposed (Figure 1a). NaAlg solution, CaCl_2_ solution, and DI water served as the inner flow, middle flow, and sheath flow, respectively. First, the inner flow and the middle flow converged in the solidifying part, and then the flow of NaAlg solution was squeezed and gradually surrounded by CaCl_2_ solution. Diffusional Ca^+^ and Alginate underwent chemical polymerization reaction at the interface between them, thus forming solidified microfiber. All the fluids in the channel are laminar flows, which ensure the stability of the flows in the channel and the stable formation of the microfibers. In the jetting part, a jetting nozzle was designed near the outlet of the channel. In order to find the appropriate parameter which can realize the jetting and have no undesirable influence on the formation of microfiber, several microfluidic chips with different parameters were fabricated and several experiments were conducted with them. Ultimately, a feasible microchannel was obtained and some design criteria were summarized according to the experimental results.

The essential parameters of jetting nozzle are illustrated in Figure 1b and Table 1. A pair of symmetric flow channels was introduced around the outlet. Particularly, $\theta_{1}$ and $\theta_{2}$ measure the angles between the sheath channel and the central channel. When $\theta_{1}$ and $\theta_{2}$ were increased, the focusing effect of the sheath flow was enhanced, but the sheath flow might flow back to the central channel and interfere with the flow of microfibers, thus clogging the channel. Therefore, all things considered, $\theta_{1}$ and $\theta_{2}$ were configured at 15° and 20° respectively in this work. Meanwhile, *d*_1_ and *d*_2_ represent the width of the central channel and the final exit. If *d*_2_ is much larger than *d*_1_, the flow velocity at the outlet will be too small to form a jet. On the other hand, if the value of *d*_2_ approaches the value of *d*_1_, DI water may flow back to the central channel again. As a result, the ratio of *d*_1_ and *d*_2_ were designed to be 1:2 in our chip. The specific value of *d*_1_ influences the formation process of the microfibers, but it has no effect on jetting performance and can thus be adjusted according to the target diameter of microfiber. A circular arc with its radius represented by *r* was used to link the sheath channel and the exit channel to prevent abrupt changes in the sheath flow. Thus, the circular arc must be tangential to the outer curve of sheath channel and the edge of the exit channel, and *r* must be as large as possible. Based on above considerations and experimental results, one set of the feasible parameters of jetting nozzle is listed in Table 1.

### 2.3. Microfluidic Device Fabrication

**Figure 2:**
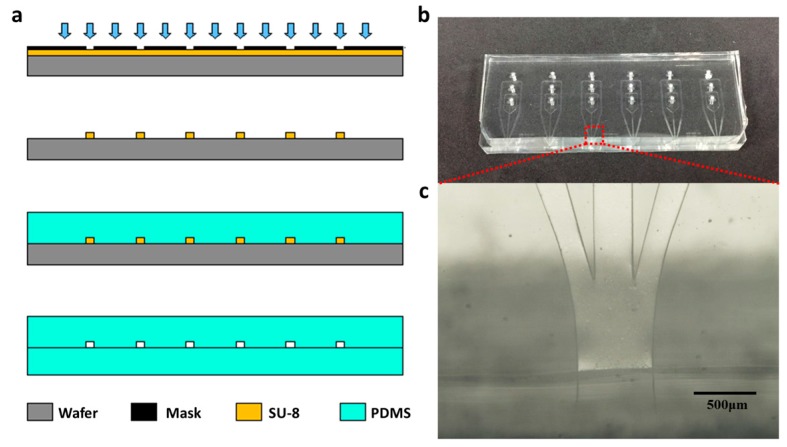
(**a**) Schematic for soft lithographic fabrication sequence; (**b**) Photo for microfluidic device with an array of microchannels which can be used individually; (**c**) The enlarged microscope image for the jetting nozzle corresponding to part in the red dotted rectangle in (**b**).

The microfluidic chip is fabricated through standard soft lithography and replica molding techniques. The specific sequences are shown in Figure 2a. First, a mask with the target microchannel pattern designed using AutoCAD software (2013 Simplified Chinese, Autodesk Inc., San Rafael, CA, USA) was made. Second, a layer of 80-μm-thick negative photoresist (SU8-2150, Microchem Co., Westborough, MA, USA) was spin-coated on a silicon wafer, and after exposure and developing, the photoresist layer with certain pattern is solidified on a wafer. The solidified photoresist layer was then used as a mold in the subsequent steps. Third, 1H,1H,2H,2H-Perfluorooctyltrichlorosilane (Apollo Scientific Ltd., Manchester, UK) was deposited on the mold to avoid adhesion in the replicating step. Fourth, a mixture of silicon elastomer base and silicon elastomer curing agent with the ratio of 10:1 was poured into the mold and then baked at 80 °C for 2 h, and then the cured PDMS that replicated the micro structured pattern of the mold was obtained. Finally, the patterned PDMS removed from the mold, was punched with inlets and bonded with another piece of flat PDMS after oxygen plasma treatment. Compared with the asymmetric structure, which was the bonding of PDMS with glass, this structure of microfluidic chip using two halves of PDMS ensured uniform hydrophilic properties on all the side walls of the microchannels. Consequently, the flow in the channel of the chip was symmetric along the thickness direction and the flow jetting from the exit was precisely vertical to the side plane of the exit. Figure 2b shows the photo of the finished microfluidic chip and Figure 2c shows the microscope image of the jetting nozzle in the microfluidic chip.

### 2.4. Fabrication and Characterization of Microfiber

The NaAlg and the CaCl_2_ solutions were separately injected into the microchannel using two syringe pumps (LSP01, Longer Co., Baoding, China). Another high flow rate syringe pump (PHD ultra, Harvard Apparatus Co., Holliston, MA, USA) was used to pump the DI water. A zoom-stereo microscope with CCD camera (Shanghai Optical Instrument Factory, Shanghai, China) was used to obtain the real-time optical image in the microchannel. After collection and sample preparation, the microfibers were observed and measured using a scanning electron microscope (Sirion 200, FEI Co., Hillsboro, OR, USA).

## 3. Results and Discussion

### 3.1. Continuous Jetting of Microfiber

**Figure 3:**
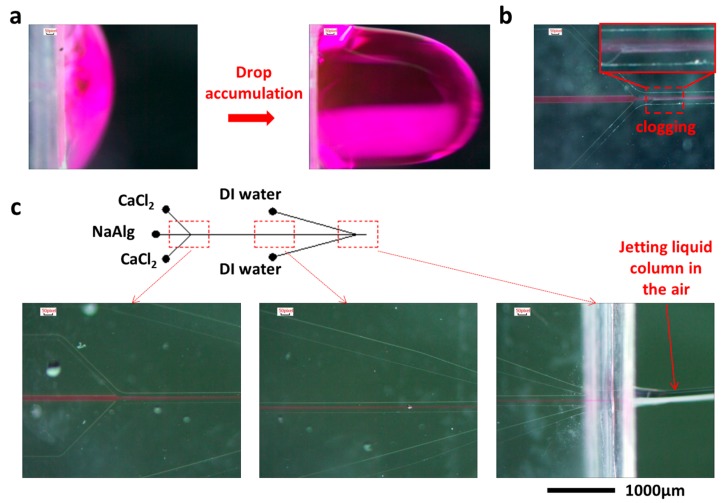
Microscope images of the microchannel during the microfiber fabrication. (**a**) Drop accumulation around the outlet of microchannel. (**b**) Drop occurrence interrupts the flow in the microchannel and thus causes clogging. The white floccule was the solidified alginate hydrogel which is stacked and clogged in the microchannel. (**c**) Optical images of microchannel when the microfiber consistently jetted from the outlet. DI water: Deionized water.

NaAlg solution, CaCl_2_ solution, and DI water were pumped into the microfluidic chip, and rhodamine B was added into the NaAlg solution to display the region where NaAlg (before solidification reaction) and fibers (after solidification reaction) existed. When the DI water was not introduced into the microchannel or its flow rate was quite low, the liquid accumulated around the outlet of the microfluidic chip, at which point the formed microfiber was restricted in the drop, as shown in Figure 3a, where the dark red object was the dyed microfiber. The accumulated drop not only affected the collection of microfibers in the outlet but also affected their formation. It brought periodic perturbations to the flow field when the drop fell down along with the trapped microfiber. In this condition, the flow rate in the channel could have been decreased because of the resistance caused by the accumulated drop, and NaAlg was excessively reacted with CaCl_2_. As a result, an irregularly shaped solidified hydrogel was formed, which caused severe clogging in the channel (Figure 3b). Increasing the flow rate of DI water gradually, though the drop still formed around the outlet, the accumulate-fall period sped up. When the flow rate of DI water exceeded 200 mL/h, the flow condition around the outlet changed from a series of drops to a stable jet, as analyzed in the principle section, and the microfiber was also jetted out with water. Figure 3c shows the microscope images of the formation of the microfibers in the channel and the stable jetting in the outlet. However, when the flow rate of DI water is increased to 500 mL/h, instability appeared again because DI water flowed back into the central channel and disturbed the flow of microfiber. After performing several trials and experiments, we obtained that the appropriate flow rate of DI water that could be used to produce stable jetting in the outlet is in the wide range of 200 to 500 mL/h. It can be concluded that the sheath flow and the designed nozzle around the outlet of the chip was effective in facilitating the jetting of microfiber and they have no significant influence on the production of microfibers as long as the flow rates were appropriate.

### 3.2. Effect of Sheath Flow on the Diameter of Microfiber

**Figure 4:**
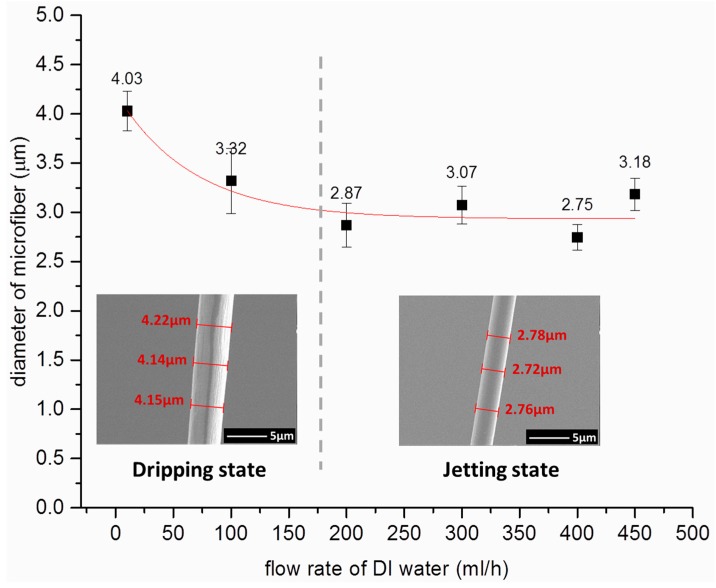
Influence of deionized (DI) water flow rate on the diameter of microfiber. The insets are the scanning electron microscope (SEM) images of uniform microfibers with different diameters.

Because the introduction of the sheath flow changed the distribution of the flow field, its influence on the morphology of microfiber must be studied. Therefore, experiments on the relationship between the flow rate of sheath flow and the diameter of microfiber were conducted. The flow rates of the NaAlg and CaCl_2_ solutions were set to 5 and 50 mL/h, respectively. Concurrently, the flow rate of DI water was successively adjusted to 0, 100, 200, 300, 400, and 500 mL/h, and several microfibers were fabricated under each of the above flow rates. The diameters of the microfibers were measured under scanning electron microscope (SEM), and the results are shown in Figure 4. A fitting curve (red curve in Figure 4) was calculated using the measurement data.

During the dripping state (the flow rate of the sheath flow ranged from 0 to 200 mL/h), the diameter of the microfiber slightly decreased when the flow rate of DI water increased. This behavior might have been caused by the introduction of the sheath flow, which accelerated the drop accumulate-fall process. The accelerated process altered the chemical reaction process, and ultimately changed the diameter of microfiber. However, when the flow condition changed to the jetting state, the diameter of microfiber was basically unchanged. The unchanged state of the microfiber could be due to the solidification of NaAlg aqueous solution (center flow) to microfibers before flowing to the jetting nozzle of the chip. Despite the pressure applied by DI water, the flow rate of the DI water had minimal influence on the diameter of the jetting microfiber. Thus, the flow rate of the DI water can be adjusted to attain ideal jetting in this range without affecting the diameter of the microfiber. However, when the flow rate of the sheath flow is relatively high (more than 500 mL/h), it may cause instability in the channel. In this situation, the microfibers cannot be collected easily and their diameter can be quite variable.

**Figure 5:**
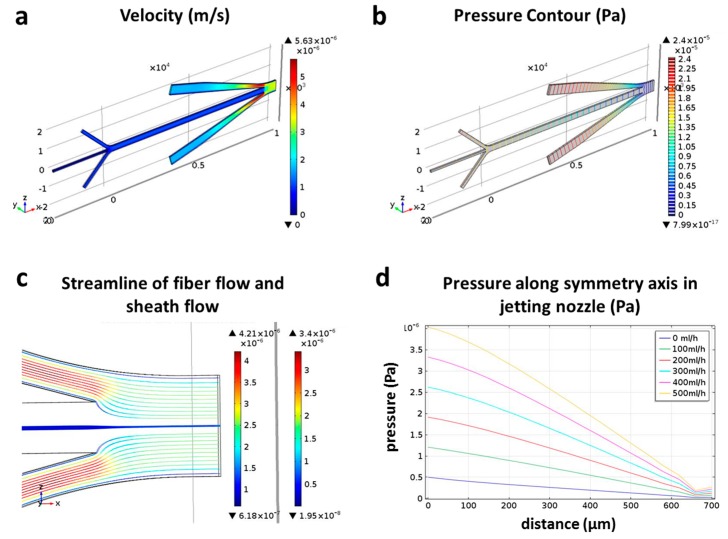
Simulation results. (**a**) Velocity distribution in the microchannel; (**b**) Pressure contour in microchannel; (**c**) Streamline of fiber flow and sheath flow; (**d**) Pressure distribution curves along symmetry axis of jetting nozzle under different flow rates of DI water.

In order to understand the internal flow field which is difficult to be characterized with experiments, computational fluid dynamic (CFD)-simulations using the software COMSOL Multiphysics 5.0 (COMSOL Inc., Stockholm, Sweden) were carried out, as presented in Figure 5. The simulation was performed on the basis of the structure of the proposed microchip and under same condition as that of the experiment: A constant flow rate of 5 mL/h for inner flow, a constant flow rate of 50 mL/h for CaCl_2_, and flow rates varying from 0 to 500 mL/h for DI water. Figure 5a,b separately show the velocity distribution and the pressure contour in the microchannel under 300 mL/h of DI water, where the water stably jets from the outlet of the chip. In particular, the velocity distribution of the inner flow and the sheath flow is shown in the Figure 5c using streamline, which demonstrates the focusing effect of sheath flow on the inner flow. Figure 5d shows the pressure distribution curves along the symmetry axis of the jetting nozzle under different flow rates of DI water. The results showed that the pressure has an approximate linear relationship with distance to the outlet, that is, the pressure gradient remained constant. But the microfiber withstands various pressures in the jetting part of the chip under different flow rates of DI water. The simulation results suggest that designing a microchannel with sufficient length for microfiber solidification is crucial for obtaining stable microfiber morphology. Actually, the length of the microchannel between the solidifying and jetting parts in the proposed microchip was enough, so the central flow had been completely solidified before flowing to the jetting part of the chip. Therefore, the experimental results showed that the DI water’s flow rate had a minimal effect on the morphology of the microfiber.

### 3.3. Microfiber Collection and Assembly

**Figure 6:**
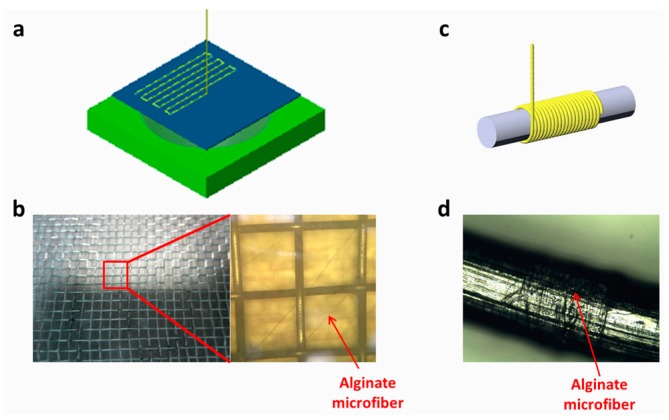
Microfiber collection and assembly. (**a**) Schematic of alginate microfiber patterning process. (**b**) Optical images of patterned alginate microfiber on a metal net substrate and the part in the red rectangle is enlarged on the right where alginate microfiber can be observed. (**c**) Schematic of alginate microfiber reeling process. (**d**) Optical image of reeled alginate microfiber on a wire.

The jetting microfiber is easy and convenient to collect and assemble. Placing a reticular structure under the outlet of the microchip as a substrate allows the microfiber to fall onto the substrate, while the redundant liquid flows past the reticular substrate. Controlling the substrate in accordance with the generation rate of the microfiber in the setting path, the microfiber can be precisely positioned on the substrate to form a certain pattern (Figure 6a,b). Furthermore, the jetting microfiber can be reeled by a roller with a rotating speed that is level with the generation rate of the microfiber (Figure 6c,d). Moreover, microfibers with different morphologies or different compositions may also be assembled through weaving or knitting. By using the jetting function and the methods above, various fiber-based macroscopic structures can be obtained.

## 4. Conclusions

Faced with challenges with regard to collection and assembly of alginate microfiber formed through chemical reaction, we constructed a simple microfluidic chip to facilitate the continuous jetting of the microfiber. Through soft lithography technology, the PDMS chip with a specially designed jetting nozzle near the exit of microchannel was fabricated. DI water was introduced into the microchannel as the sheath flow through the jetting nozzle, thus successfully preventing the dripping state around the outlet of microchannel. The experiments showed that the nozzle and the use of sheath flow effectively facilitated continuous jetting of the microfiber and the enhancement of the flow field stability. Moreover, in microfibers under continuous jetting, the specific flow rate of DI water had no influence on the morphology of microfiber, which can be explained by complete solidification of the microfiber before it reached the jetting nozzle. The jetting microfiber can be conveniently collected and assembled into complicated structures through various methods, such as patterning, reeling, and weaving. This microfluidic method is rather simple and the jetting nozzle can be easily integrated into other structures without additional production steps. Therefore, it can be applied for synthesizing microfiber and construct fiber-based macroscopic structures in the fields of biotechnology and tissue engineering.

## Figures and Tables

**Table 1:** Parameters and their specific values of jetting nozzle.

Parameters	Definition	Values
$\theta_{1}$	Angle between the inner edge curve of sheath channel and the edge line of the central channel	15°
$\theta_{2}$	Angle between the outer edge curve of sheath channel and the edge line of the central channel	20°
*d*_1_	Width of central channel	300 μm
*d*_2_	Width at the outlet	600 μm
*r*	Radius of nozzle	3000 μm
*h*	Height of microfluidic channel	80 μm
